# Prediction of desmoglein-3 peptides reveals multiple shared T-cell epitopes in HLA DR4- and DR6- associated Pemphigus vulgaris

**DOI:** 10.1186/1471-2105-7-S5-S7

**Published:** 2006-12-18

**Authors:** Joo Chuan Tong, Tin Wee Tan, Animesh A Sinha, Shoba Ranganathan

**Affiliations:** 1 Department of Biochemistry, Yong Loo Lin School of Medicine, National University of Singapore, 8 Medical Drive, Singapore 117597, Singapore; 2 Institute for Infocomm Research, 21 Heng Mui Keng Terrace, Singapore 119613, Singapore; 3 Center for Investigative Dermatology, Division of Dermatology and Cutaneous Sciences, College of Human Medicine, Michigan State University, 4120 Biomedical and Physical Sciences Building, East Lansing, MI 48824, USA; 4 Department of Chemistry and Biomolecular Sciences & Biotechnology Research Institute, Macquarie University, Sydney NSW 2109, Australia

## Abstract

**Background:**

Pemphigus vulgaris (PV) is a severe autoimmune blistering skin disorder that is strongly associated with major histocompatibility complex class II alleles DRB1*0402 and DQB1*0503. The target antigen of PV, desmoglein 3 (Dsg3), is crucial for initiating T-cell response in early disease. Although a number of T-cell specificities within Dsg3 have been reported, the number is limited and the role of T-cells in the pathogenesis of PV remains poorly understood. We report here a structure-based model for the prediction of peptide binding to DRB1*0402 and DQB1*0503. The scoring functions were rigorously trained, tested and validated using experimentally verified peptide sequences.

**Results:**

High predictivity is obtained for both DRB1*0402 (*r*^2 ^= 0.90, *s *= 1.20 kJ/mol, *q*^2 ^= 0.82, *s*_*press *_= 1.61 kJ/mol) and DQB1*0503 (*r*^2 ^= 0.95, *s *= 1.20 kJ/mol, *q*^2 ^= 0.75, *s*_*press *_= 2.15 kJ/mol) models, compared to experimental data. We investigated the binding patterns of Dsg3 peptides and illustrate the existence of multiple immunodominant epitopes that may be responsible for both disease initiation and propagation in PV. Further analysis reveals that DRB1*0402 and DQB1*0503 may share similar specificities by binding peptides at different binding registers, thus providing a molecular mechanism for the dual HLA association observed in PV.

**Conclusion:**

Collectively, the results of this study provide interesting new insights into the pathology of PV. This is the first report illustrating high-level of cross-reactivity between both PV-implicated alleles, DRB1*0402 and DQB1*0503, as well as the existence of a potentially large number of T-cell epitopes throughout the entire Dsg3 extracellular domain (ECD) and transmembrane region. Our results reveal that DR4 and DR6 PV may initiate in the ECD and transmembrane region respectively, with implications for immunotherapeutic strategies for the treatment of this autoimmune disease.

## Background

Pemphigus vulgaris (PV) is a severe autoimmune blistering skin disorder due to loss of integrity of normal intercellular attachments within the epidermis and mucosal epithelium. Strong association of PV to the major histocompatibility complex (MHC) class II alleles DRB1*0402 and DQB1*0503 have been reported in the literature [1-6] with over 95% of PV patients possessing one or both of these alleles [7,8]. The molecular basis and immunological consequences of this dual HLA association has thus far not been elucidated. The target antigen of PV, desmoglein (Dsg) 3, is a 130-kDa transmembrane glycoprotein that belongs to the cadherin superfamily of cell adhesion molecules [9]. In the early stage of disease, patients demonstrate autoimmunity only to Dsg3 and develop mucosal blisters; while at the later stage, patients exhibit non-cross-reactive immunity to both Dsg3 and Dsg1 [10]. Despite several reports of T-cell specificities within Dsg3 [11-16], the number is limited and the role of T-cells in the pathogenesis of PV remains poorly understood.

Bioinformatic tools are now commonly used in facilitating T-cell epitope discovery [17-19]. Computational methods for predicting MHC-binding peptides include procedures based on sequence motifs [13,16], quantitative matrices [20-22], decision trees [23,24], artificial neural networks [25,26], hidden Markov models [27] and support vector machines [28-30]. However, despite recent advances in sequence-based predictive techniques, effective models for DRB1*0402 and DQB1*0503 have been lacking, mainly due to the paucity of sufficient peptides as training data [31] as well as register-shifts (or positional differences between the core nonameric peptide in the binding groove) [31] and polymorphisms in the binding registers (A.A. Sinha, unpublished results). To date, few prediction techniques for MHC class II molecules have been developed using three-dimensional models as the dual issues of model quality and discrimination technique must be addressed [32].

Our strategy for prediction of T-cell epitopes involves the three-dimensional modeling of peptide/MHC complexes using a hybrid docking approach that integrates the strengths of Monte Carlo simulations and homology modeling [31,33,34]. In an earlier study, we have successfully discriminated disease-implicated from non-disease implicated and protective alleles in PV based on structural interaction rules [31]. A complementary scoring function has now been developed for effective identification of DRB1*0402 and DQB1*0503 epitopes. We investigated the T-cell epitope repertoire of the entire Dsg3 glycoprotein and show the existence of multiple extracellular and intracellular specificities within the Dsg3 self-antigen. Further analysis reveal that DRB1*0402 and DQB1*0503 share similar specificities by binding peptides at different core recognition regions. These data provide a molecular rationale for the association of PV to two distinct HLA molecules and impact our understanding of the mechanism of HLA mediated control of disease.

## Results and Discussion

### DRB1*0402 predictive model

The DRB1*0402 model shows excellent correlation with experimental binding affinities (*r*^2 ^= 0.90, *s *= 1.20 kJ/mol, *q*^2 ^= 0.82, *s*_*press *_= 1.61 kJ/mol). The accuracy of the prediction model was further evaluated using (i) peptides with experimental IC_50 _values obtained from biochemical studies and (ii) Dsg3 peptides with T-cell proliferation values obtained from functional studies.

Three threshold binding energy values (Table 1) that define levels of specificities suitable for practical applications [34] were used to evaluate the accuracy of the DRB1*0402 prediction model on the biochemical dataset (Test set 1) – LMH (low-, medium-, high-affinity binders; A_ROC _= 0.93); MH (medium- and high-affinity binders; A_ROC _= 0.86) and H (high-affinity binders only; A_ROC _= 0.81). The results indicate that, overall, our DRB1*0402 peptide-binding models are highly accurate (A_ROC _= 0.93). SP = 0.80 offers high-sensitivity predictions, whereas SP = 0.95 results in very few false positives but fewer true positives. The prediction results for our model were consistent with expected binding patterns of DRB1*0402 peptides and provided a sensitivity of 70% (SP = 0.95) for DRB1*0402-binding peptides.

Next, the predictive performance of the DRB1*0402 model was tested on the functional dataset of 25 peptides (Test set 2) with experimental T-cell proliferation values/responses using the decision thresholds defined above. All experimental positives (Table 2) are predicted with binding energy values of -26.64 kJ/mol or less (high-affinity binders; SE = 0.65, SP = 0.80). Our simulation results indicate that DRB1*0402 can bind Dsg3 96–112 at threshold -26.64 kJ/mol (SE = 0.65, SP = 0.80) in contrast to previous qualitative study results [31], albeit at a binding energy (-27.09 kJ/mol) close to the threshold. Dsg3 512–526 (ranked #10), Dsg3 78–93 (ranked #12) and Dsg3 96–110 (ranked #19) are predicted binders (SE = 0.65, SP = 0.80) that did not stimulate T-cell responses in the relevant experiments [11,13,16]. Noteworthy, peptides Dsg3 78–93 [13] and Dsg3 96–110 [16] are fully contained within Dsg3 78–94 (ranked #11) and Dsg3 96–112 (ranked #22), both of which are experimental true positives identified in an independent study [11]. For Dsg3 78–93, the preferred binding register identified by our models is 79–87, which is shows a 5-residue shift in the binding register from the preferred core of 82–90 for Dsg3 78–94. There is no change in the binding register observed for Dsg3 96–110 and Dsg3 96–112. It is possible that variability in the binding register (#12) or flanking peptide residues (#19) lead to a change in T-cell stimulation.

### DQB1*0503 predictive model

The DQB1*0503 prediction model correlate well with experimental data (*r*^2 ^= 0.95, *s *= 1.20 kJ/mol). The DQB1*0503 model outperforms the prediction models done by Rognan *et al*. (1999) on training datasets of 5 HLA-A*0204 (*r*^2 ^= 0.85, *s*_*press *_= 2.40 kJ/mol) and 37 H2-K^k ^(*r*^2 ^= 0.78, *s*_*press *_= 3.16 kJ/mol) peptide sequences and is consistent with our DRB1*0402 prediction model (*r*^2 ^= 0.90, *s *= 1.20 kJ/mol, *q*^2 ^= 0.82, *s*_*press *_= 1.61 kJ/mol) which is trained using the same number of data points. The cross-validation coefficient *q*^2 ^and the standard error of prediction *s*_*press *_are stable, with *q*^2 ^= 0.75 and *s*_*press *_= 2.15 kJ/mol. This iterative regression procedure validates the internal consistency of the scoring function in the current model, rendering it suitable for predictions on the test dataset obtained from functional studies.

The accuracy of the DQB1*0503 prediction model was assessed on a dataset of 6 (5 stimulatory and 1 non-stimulatory peptides) Dsg3 peptides with known T-cell proliferation values (Table 3). All DQB1*0503-specific Dsg3 stimulatory peptides can be effectively discriminated from the background at the prediction threshold -26.64 kJ/mol.

### Disease progression in PV

**Figure 1:**
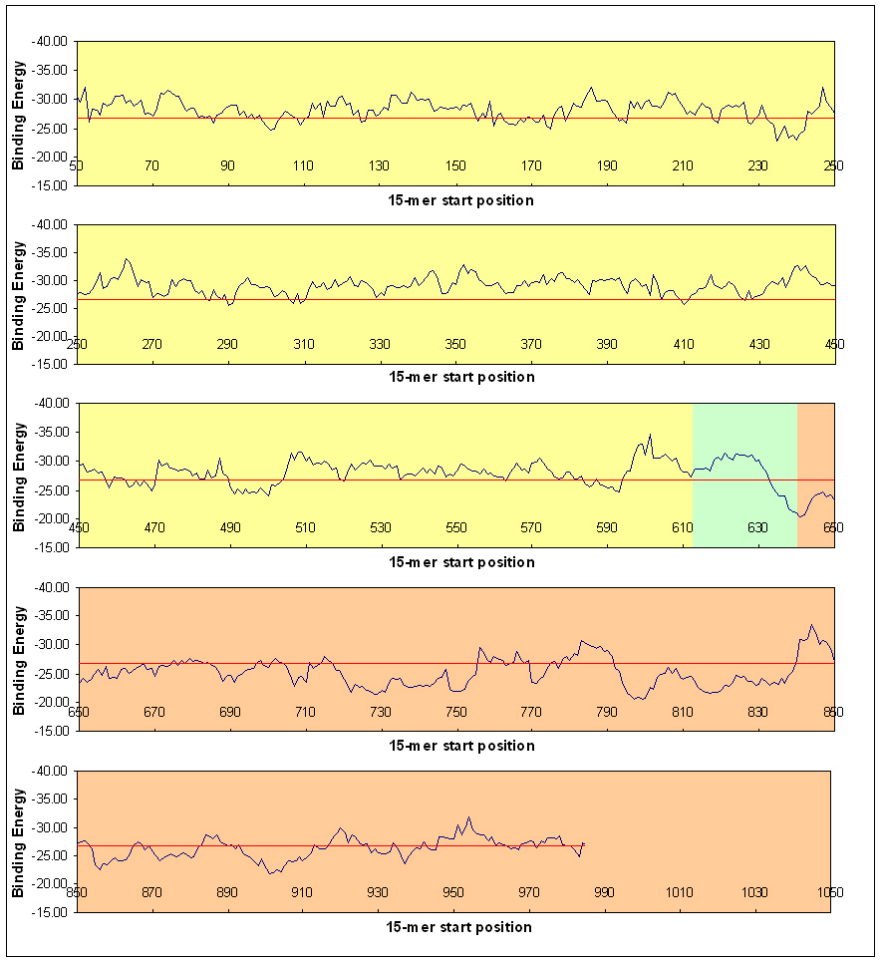
Predicted DRB1*0402-specific T-cell epitope repertoire within Dsg3. Location of predicted DRB1*0402-specific T-cell epitopes along the Dsg3 glycoprotein – extracellular domain (colored yellow; Dsg3 50–615), transmembrane region (colored green; Dsg3 616–640), and intracellular domain (colored pink; Dsg3 641–999). The red line indicates decision threshold -26.64 kJ/mol for high-affinity binders (SP = 0.80, SE = 0.65).

**Figure 2:**
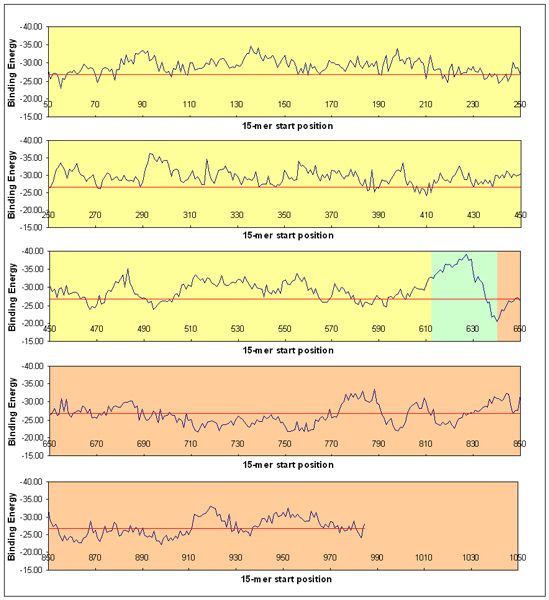
Predicted DQB1*0503-specific T-cell epitope repertoire within Dsg3. Location of predicted DQB1*0503-specific T-cell epitopes along the Dsg3 glycoprotein – extracellular domain (colored yellow; Dsg3 50–615), transmembrane region (colored green; Dsg3 616–640), and intracellular domain (colored pink; Dsg3 641–999). The red line indicates decision threshold -26.64 kJ/mol for high-affinity binders (SP = 0.80, SE = 0.65).

A variety of studies have demonstrated that a limited set of epitopes may be present in early disease, and intra-molecular epitope spreading may occur during disease transition at the B-cell level [10]. Our data support the existence of multiple immunodominant T-cell epitopes that may be responsible for both disease initiation and propagation (Figure 1 and Figure 2). These findings are in line with T-cell proliferation data obtained from DR4 and DR6 PV patients [11,13,15,16,31]. Our analysis showed that the potential Dsg3 T-cell epitope repertoire is well distributed throughout all five extracellular domains (ECDs) (ECD1: Dsg3 50–158; ECD2: Dsg3 159–268; ECD3: Dsg3 269–383; ECD4: Dsg3 386–499; ECD5: Dsg3 500–615) [35]. A large number of DQB1*0503-specific Dsg3 peptides were predicted to exist within the transmembrane region (Dsg3 616–640; Figure 2). Noteworthy, 13 of these peptides were found among the top 20 predictions (Table 4), suggesting that disease initiation may begin at the transmembrane region for DR6 PV patients. Such an initiation step would not be unique to PV as other studies have revealed disease initiation at the transmembrane region [36,37]. For DRB1*0402, 90% (18/20) of the top 20 predictions were predicted to exist within the ECDs, suggesting that disease initiation in DR4 and DR6 PV patients may be different. Although two DRB1*0402-specific intracellular peptides (Dsg3 762–786, Dsg3 786–800) have been reported [13,16], and were correctly detected by our model (SP = 0.80, SE = 0.65), relatively few intracellular peptides from the region, Dsg3 641–999, are predicted by our methods. It remains to be determined what proportion of the predicted epitopes are actually generated via antigen processing events *in vivo*, or remain subdominant or "cryptic", such that they are not available for recognition during the initial immune response. It is likely that immune responses develop against secondary epitopes at later stages of disease progression, as a result of intramolecular epitope spreading, and it will be important to delineate the cascade of disease relevant epitopes in temporal sequence in future work.

### DRB1*0402 and DQB1*0503 cross reactivity

An in-depth analysis was performed to investigate the extent of overlap in the Dsg3 peptide-binding repertoires of DRB1*0402 and DQB1*0503. A panel of 936 15mer Dsg3 sequences were generated using an overlapping sliding window of size 15 across the entire Dsg3 glycoprotein and modeled into the binding grooves of both DRB1*0402 and DQB1*0503 (explained in detail in *Peptide docking*).

Both the DRB1*0402 and DQB1*0503 alleles are particularly efficient in binding Dsg3-derived peptides. Furthermore, a significant level of cross-reactivity was observed between DRB1*0402 and DQB1*0503. Of the 936 overlapping 15mer peptides generated from the entire Dsg3 glycoprotein investigated in this study, 539 (57%) were predicted high-affinity binders to both alleles at threshold -26.64 kJ/mol. The computer simulation results are shown in Figures 1 and 2. Noteworthy, three previously defined immunoreactive segments of the Dsg3 extracellular domains (Dsg3 145–192, 240–303 and 570–614) [38] were also predicted by our models at this specific threshold. Among the known Dsg3 peptides (Tables 2 and 3), only 2 (Dsg3 191–205 and 762–776) were predicted to bind DRB1*0402 alone, consistent with our earlier qualitative results from structural studies alone [31]. 18 (Dsg3 78–94, 96–112, 161–177, 189–205, 190–204, 205–221, 206–220, 210–226, 250–266, 251–265, 342–356, 342–358, 376–392, 380–396, 483–499, 786–800, 810–824, 963–977) were predicted to bind to both DRB1*0402 and DQB1*0503. These observations are of particular interest in that both DRB1*0402 and DQB1*0503 are strongly linked to PV [39,40], indicating that common or overlapping dominant epitopes may be responsible for inducing disease in DR4 and DR6 patients respectively.

### DRB1*0402 and DQB1*0503 peptide binding specificities

**Figure 3:**
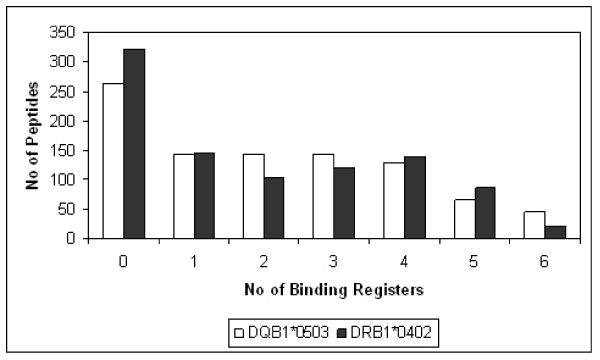
Predicted binding registers for Dsg3 peptides bound to DRB1*0402 and DQB1*0503. The frequency of Dsg3 peptides docked to DRB1*0402 (colored gray) and DQB1*0503 (colored white) is shown as a function of the number of predicted binding registers.

The basis for the high degree of cross-reactivities between DRB1*0402 and DQB1*0503 was subjected to further analysis. Our data support the existence of multiple binding registers within a candidate binding peptide that serve as recognition sites for DRB1*0402 and DQB1*0503, an observation previously noted for DQB1*0302 binding peptides [34]. Of 936 Dsg3 sequences, 614 were predicted high-affinity binders with 76% displaying 2 or more registers that can be docked into the binding groove of DRB1*0402 (Figure 3). Similar results are obtained for DQB1*0503, with 673 predicted high-affinity binders and 57% exhibiting 2 or more binding registers (Figure 3). DRB1*0402 and DQB1*0503 predicted consensus binding sequences number 539. A striking aspect of this analysis is that DRB1*0402 and DQB1*0503 were predicted to bind a large portion of these peptides (354/539 or 66%) at different binding registers. Noteworthy, this difference was detected in 70% (7/10) of Dsg3 peptides known to bind both DRB1*0402 and DQB1*0503 (Table 5). For example, the consensus binding peptide Dsg 205–221 showed ΔG values less than the decision threshold -26.64 kJ/mol, with the 211–219 and 212–220 registers being the preferred binding modes for DRB1*0402 and DQB1*0503 respectively. We propose that DRB1*0402 and DQB1*0503 share similar specificities by binding peptides at different binding registers.

## Conclusion

Although PV was first reported by Hippocrates in 400 B.C., few T-cell specificities within Dsg3 have been identified to date [11-16], and the role of MHC and T-cells in the pathogenesis of PV still remains poorly understood. Collectively, the results of this study provide interesting new insights into the pathology of PV. This is the first report of high-level of cross-reactivity between both PV-implicated alleles, DRB1*0402 and DQB1*0503, as well as the existence of a potentially large number of T-cell epitopes throughout the entire Dsg3 ECD and transmembrane region. Our data strongly indicates that multiple initial epitopes may be responsible for both disease initiation and progression. In addition, our modelling results revealed that DR4 and DR6 PV may initiate at different regions of Dsg3. DR4 PV may initiate in the ECD while DR6 PV may begin at the transmembrane region. It remains to be determined what proportion of predicted DRB1*0402 and DQB1*0503 binders are capable of stimulating PV-implicated alleles and autoreactive T-cells. If experimental analysis reveals limited sets of predicted Dsg3 peptides capable of eliciting functional responses, control over autoreactivity may rest at the T-cell level, rather than the level of determinant selection by MHC molecules. This will have a direct impact in the design of immunotherapeutic strategies for the treatment of this autoimmune disease.

## Methods

MHC sequence data were obtained from IMGT-HLA database [41]. To identify potential structural templates available in the Protein Data Bank (PDB) [42] for model building, a sequence similarity search was performed using PSI-BLAST [43] running on the servers at NCBI and the highest quality templates (with the best resolution, highest sequence similarity and minimal number of missing residues) were selected among the returned results. The crystal structures of DRB1*0401 (PDB code 1D5Z) and DQB1*0602 (PDB code 1UVQ) were selected as templates for DRB1*0402 (97.9% identity) and DQB1*0503 (93.0% identity) respectively.

### Model building

The program MODELLER [44] was employed for comparative modeling of both DRB1*0402 and DQB1*0503. The models are constructed by optimally satisfying spatial constraints obtained from the alignment of the template structure with the target sequence and from the CHARMM-22 force field [45]. The structures were relaxed by conjugate gradient minimization, using the Internal Coordinate Mechanics (ICM) 3.0 package [46].

### Experimental binding data

Two sets of data are used in this study: (i) peptides with experimental IC_50 _values from biochemical studies and (ii) peptides with experimental T-cell proliferation values/responses from functional studies.

Dataset I (Additional file 1: Table S1) comprises 59 DRB1*0402-specific peptides derived from biochemical studies (20 high-affinity, 11 medium-affinity and 13 low-affinity binders and 15 non-binders). Peptides are classified based on their experimental IC_50 _values (high-affinity binders: IC_50 _≤ 500 nM, medium-affinity binders: 500 nM < IC_50 _≤ 1500 nM, low-affinity binders: 1500 < IC_50 _≤ 5000 nM and non-binders: 5000 < IC_50_).

Dataset II (Additional file 2: Table S2) consists of 25 DRB1*0402-specific Dsg3 peptides and 14 DQB1*0503-specific Dsg3 peptides with T-cell proliferation values/responses [11-13].

### Peptide docking

Overlapping 15-mer peptides are generated from the Dsg3 sequence. An overlapping sliding window of size nine is applied to each 15-mer peptide to generate all combinations of binding registers to be modeled into the binding grooves of DRB1*0402 and DQB1*0503. Docking was performed with the Empirical Conformational Energy Program for Peptides 3 force field parameters (ECEPP/3) [46] and MMFF partial charges [47] on a 4-CPU SGI Origin 3200 workstation using an extension of the protocol [33,34]: (i) pseudo-Brownian rigid body docking of peptide fragments to the ends of the binding groove, (ii) central loop closure by satisfaction of spatial constraints, (iii) refinement of the backbone and side-chain atoms of the core recognition residues and receptor contact regions within 4.00Å radius [31,33] and (iv) extension of flanking peptide residues by satisfaction of spatial constraints.

### Empirical free energy functions

The scoring function presented in this study is based on the free energy potential in ICM3.0 package [47]. The binding free energy function is partitioned into three terms [34] expressed by the equation:

ΔG = αΔG_H _+ βΔG_S _+ γΔG_EL _+ *C*.     (1)

ΔG_H _is the hydrophobic energy computed as the product of solvent accessible surface area (determined by rolling a sphere of 1.40Å radius along the surface of the molecule) by the surface tension. ΔG_S _refers to the entropic contribution from the protein side-chains computed from the maximal burial entropies for each type of amino acid and their relative accessibilities. ΔG_EL _denotes the electrostatic term composed of coulombic interactions between receptor and ligand and the desolvation of partial charges transferred from an aqueous medium to a protein core environment, and is determined by the numeric solution of the Poisson equation using an implementation of the boundary element algorithm [48-50]. The constant term *C *accounts for entropy change in the system due to the decrease of free molecular concentration and the loss of rotational/translational degrees of freedom upon binding [51].

### Training, testing and validation

Two computational models are trained in this study – one model for the prediction of peptide binding to DRB1*0402 and the other for DQB1*0503.

DRB1*0402-specific peptide data derived from biochemical studies with experimental IC_50 _values was divided into training and test sets. The training set comprised 8 (5 binding and 3 non-binding) Dsg3 sequences with experimentally determined binding registers (from Dataset I). Two external sets of test data were used: (i) Test set 1: 51 peptides with experimental IC_50 _values (20 high-affinity binders, 11 medium affinity binders, 9 low affinity binders and 11 non-binders) from biochemical studies, and (ii) Test set 2: all DRB1*0402-specific Dsg3 peptides from Dataset II, with known T-cell proliferation values.

DQB1*0503 prediction model was trained using Dsg3 peptide data from functional studies in the absence of relevant biochemical data. The training set comprised 8 (5 stimulatory and 3 non-stimulatory) sequences from Dataset II. For each peptide sequence, T-cell proliferation value [13] is mapped to a theoretical IC_50 _value in accordance with expected binding patterns of Dsg3 binding peptides (Sinha *et al*., personal communications). The performance of the prediction model was subsequently evaluated on an external set of 6 peptides with known T-cell proliferation values.

Coefficients (α, β, γ) and the constant term *C *in Equation 1 were derived using standard least-square multivariate regression analyses of the training set, followed by leave-one-out analysis to assess to quality of the scoring function [52]. For each model, the entire procedure is repeated 8 times to reduce noises in all computations, the results averaged and the observed error rate is used to estimate the expected error rate upon generalization to new data.

The optimal scoring function selected from each cross-validation analysis was further assessed using sensitivity (SE), specificity (SP) and receiver operating characteristic (ROC) analysis [34]. SE = TP/(TP+FN) and SP = TN/(TN+FP), indicate percentages of correctly predicted binders and non-binders, respectively. TP (true positives) represents experimental binders with at least one predicted binding register and TN (true negatives) for experimental non-binders with no predicted binding register. FN (false negatives) denotes experimental binders predicted as non-binders and FP (false positives) represents experimental non-binders predicted as binders. The accuracy of our predictions was assessed by ROC analysis where the ROC curve is generated by plotting SE as a function of (1-SP) for various classification thresholds. The area under the ROC curve (A_ROC_) provides a measure of overall prediction accuracy, A_ROC _<70% for poor, A_ROC_>80% for good and A_ROC_>90% for excellent predictions [34]. In this study, we assessed SE for three values of SP (80%, 90% and 95%) that are considered useful in practice. All regression and validation results, including correlation coefficient (*r*^2^), standard deviation (*s*), cross-validation coefficient (*q*^2^), standard error of prediction (*s*_press_), SE, SP, A_ROC_, coefficients and constant terms in the scoring function, are recorded.

## Authors' contributions

JCT carried out the computational simulation studies and drafted the manuscript. AAS carried out the immunoassays. TWT, AAS and SR participated in the design of the study and interpretation of data. SR developed the project and finalized the manuscript.

## Supplementary Material

Additional file 1 Table-S1 Click here for file

Additional file 2 Table-S2 Click here for file

## Figures and Tables

**Table 1:** Prediction accuracy of the DRB1*0402 peptide-binding model.

**Specificity (SP) Level**	**Group**	**Sensitivity (SE)**	**Binding Energy Threshold (kJ/mol)**
SP = 0.80	LMH	0.78	-25.55
	MH	0.81	-25.79
	H	0.65	-26.64
SP = 0.90	LMH	0.75	-25.79
	MH	0.52	-26.94
	H	0.30	-28.83
SP = 0.95	LMH	0.70	-26.94
	MH	0.42	-27.72
	H	0.25	-30.57

Sensitivity (SE) values and binding energy thresholds for DRB1*0402 peptide-binding model at specificity (SP) levels 0.80, 0.90 and 0.95.

**Table 2:** Predicted binding affinities of Dsg3 peptide sequences to DRB1*0402.

**Rank**	**Peptide**	**Sequence**	**Predicted BE (kJ/mol)**	**References**
1	Dsg3 342–358	SVKLSIAVKNKAEFHQS	-31.46	Veldman *et al*. (2004)
2^#^	Dsg3 342–356	SVKLSIAVKNKAEFH	-30.88	Moesta *et al*. (2002)
3	Dsg3 205–221	GTPMFLLSRNTGEVRTL	-30.44	Veldman *et al*. (2004)
4* ^#^	Dsg3 846–860	LDSLGPKFKKLAEIS	-30.17	Moesta *et al*. (2002)
5	Dsg3 380–396	GIAFRPASKTFTVQKGI	-29.97	Riechers *et al*. (1999)
6^@^	Dsg3 67–81	RNPIAKITSDYQATQ	-29.94	Moesta *et al*. (2002)
7	Dsg3 786–800	MNFLDSYFSQKAFAC	-29.78	Moesta *et al*. (2002)
8	Dsg3 190–204	LNSKIAFKIVSQEPA	-29.74	Wucherpfennig *et al*. (1995)
9	Dsg3 189–205	HLNSKIAFKIVSQEPAG	-29.24	Veldman *et al*. (2004)
10*	Dsg3 512–526	SARTLNNRYTGPYTF	-29.21	Wucherpfennig *et al*. (1995)
11	Dsg3 78–94	QATQKITYRISGVGIDQ	-28.37	Veldman *et al*. (2004)
12*	Dsg3 78–93	QATQKITYRISGVGID	-28.30	Wucherpfennig *et al*. (1995)
13	Dsg3 191–205	NSKIAFKIVSQEPAG	-28.16	Moesta *et al*. (2002)
14	Dsg3 206–220	TPMFLLSRNTGEVRT	-28.02	Wucherpfennig *et al*. (1995)
15	Dsg3 210–226	LLSRNTGEVRTLTNSL	-27.98	Veldman *et al*. (2004)
16	Dsg3 251–265	CECNIKVKDVNDNFP	-27.88	Wucherpfennig *et al*. (1995)
17	Dsg3 250–266	QCECNIKVKDVNDNFPM	-27.68	Veldman *et al*. (2004)
18	Dsg3 483–499	VRVPDFNDNCPTAVLEK	-27.48	Veldman *et al*. (2004)
19* ^@^	Dsg3 96–110	PFGIFVVDKNTGDIN	-27.47	Moesta *et al*. (2002)
20	Dsg3 762–776	QSGTMRTRHSTGGTN	-27.28	Wucherpfennig *et al*. (1995)
21	Dsg3 161–177	IFMGEIEENSASNSLVM	-27.09	Hertl *et al*. (1998)
22	Dsg3 96–112	PFGIFVVDKNTGDINIT	-27.09	Veldman *et al*. (2004)
23^@^	Dsg3 963–977	ERVICPISSVPGNLA	-26.87	Moesta *et al*. (2002)
24*	Dsg3 97–111	FGIFVVDKNTGDINI	-26.58	Wucherpfennig *et al*. (1995)
25^@^	Dsg3 810–824	NDCLLIYDNEGADAT	-24.00	Moesta *et al*. (2002)

Predicted binding affinities of Dsg3 peptide sequences to DRB1*0402. '^#^', '^@^', '*' indicate binding, non-binding and non-immunostimulatory in the relevant experiments respectively.

**Table 3:** Predicted binding affinities of Dsg3 peptide sequences to DQB1*0503.

**Rank**	**Peptide**	**Sequence**	**Predicted BE (kJ/mol)**	**References**
1	Dsg3 206–220	TPMFLLSRNTGEVRT	-30.53	Wucherpfennig *et al*. (1995)
2	Dsg3 189–205	HLNSKIAFKIVSQEPAG	-29.10	Veldman *et al*. (2004)
3	Dsg3 78–94	QATQKITYRISGVGIDQ	-27.14	Veldman *et al*. (2004)
4	Dsg3 190–204	LNSKIAFKIVSQEPA	-26.88	Wucherpfennig *et al*. (1995)
5	Dsg3 251–265	CECNIKVKDVNDNFP	-26.65	Wucherpfennig *et al*. (1995)
6*	Dsg3 762–786	QSGTMRTRHSTGGTNKDYADGAISM	-22.42	Wucherpfennig *et al*. (1995)

Predicted binding affinities of Dsg3 peptide sequences to DQB1*0503. '*' indicates non-immunostimulatory in the relevant experiments.

**Table 4:** Top 20 predicted Dsg3 peptide sequences binding to DQB1*0503 and DRB1*0402.

**Rank**	DQB1*0503			DRB1*0402		
		
	**Peptide**	**Sequence**	**Predicted B.E. (kJ/mol)**	**Peptide**	**Sequence**	**Predicted B.E. (kJ/mol)**
1	Dsg3 627–641	GLLLL**LLAPLLLLT**C	-39.23	Dsg3 601–615	TT**SPGTRYGRP**HSGR	-34.64
2	Dsg3 626–640	LGLL**LLLLAPLLL**LT	-38.43	Dsg3 263–277	N**FPMFRDSQY**SARIE	-33.86
3	Dsg3 624–638	LLLG**LLLLLLAPL**LL	-37.87	Dsg3 844–858	S**FLDSLGPKF**KKLAE	-33.46
4	Dsg3 629–643	LLL**LLAPLLLLT**CDC	-37.82	Dsg3 264–278	F**PMFRDSQYS**ARIEE	-33.22
5	Dsg3 625–639	LLGL**LLLLLAPLL**LL	-37.65	Dsg3 599–613	Y**PTTSPGTRY**GRPHS	-33.10
6	Dsg3 628–642	LLLL**LLAPLLLLT**CD	-37.47	Dsg3 352–366	KAEFHQ**SVISRYRVQ**	-32.88
7	Dsg3 620–634	AIG**LLLLGLLLL**LLA	-36.48	Dsg3 598–612	S**YPTTSPGTR**YGRPH	-32.76
8	Dsg3 623–637	LLLLGL**LLLLLAPLL**	-36.44	Dsg3 442–456	AEIKF**VKNMNRDST**F	-32.60
9	Dsg3 618–632	PA**AIGLLLLGL**LLLL	-36.36	Dsg3 440–454	KTA**EIKFVKNMN**RDS	-32.56
10	Dsg3 293–307	LD**EEYTDNWLA**VYFF	-36.28	Dsg3 439–453	SKT**AEIKFVKNM**NRD	-32.36
11	Dsg3 622–636	GLLL**LGLLLLLLA**PL	-36.23	Dsg3 52–66	VKFAK**PCREGEDNS**K	-32.13
12	Dsg3 619–633	AA**IGLLLLGLL**LLLL	-36.20	Dsg3 186–200	EPN**HLNSKIAFK**IVS	-32.10
13	Dsg3 294–308	DE**EYTDNWLAV**YFFT	-36.15	Dsg3 354–368	E**FHQSVISRY**RVQST	-32.10
14	Dsg3 621–635	IGLLL**LGLLLLLLA**P	-35.67	Dsg3 247–261	LSTQC**ECNIKVKDV**N	-32.08
15	Dsg3 297–311	YTDNWL**AVYFFTSGN**	-35.28	Dsg3 262–276	DNF**PMFRDSQYS**ARI	-32.07
16	Dsg3 483–497	VRVP**DFNDNCPTA**VL	-35.17	Dsg3 845–859	F**LDSLGPKFK**KLAEI	-32.07
17	Dsg3 616–630	LGPA**AIGLLLLGL**LL	-34.86	Dsg3 954–968	LL**TQNVIVTER**VICP	-31.93
18	Dsg3 317–331	EIQTD**PRTNEGILK**V	-34.75	Dsg3 351–365	NKA**EFHQSVISR**YRV	-31.87
19	Dsg3 295–309	EEY**TDNWLAVYF**FTS	-34.67	Dsg3 344–358	K**LSIAVKNKA**EFHQS	-31.85
20	Dsg3 136–150	DVEK**PLILTVKIL**DI	-34.63	Dsg3 441–455	TA**EIKFVKNMN**RDST	-31.72

The nonameric core peptide in the binding groove is shown in bold, underlined text.

**Table 5:** Preferred binding registers of experimentally determined Dsg3 peptides for PV implicated alleles.

**Peptide**	**Allele**	**Sequence**	**Peptide**	**Allele**	**Sequence**
Dsg3 78–94	DRB1*0402	QA**TQKITYRIS**GVGIDQ	Dsg3 250–266	DRB1*0402	QC**ECNIKVKDV**NDNFPM
	DQB1*0503	QATQ**KITYRISGV**GIDQ		DQB1*0503	QCECNIK**VKDVNDNFP**M
Dsg3 96–112	DRB1*0402	PFGI**FVVDKNTGD**INIT	Dsg3 251–265	DRB1*0402	C**ECNIKVKDV**NDNFP
	DQB1*0503	PFGIFV**VDKNTGDIN**IT		DQB1*0503	CECNIK**VKDVNDNFP**
Dsg3 161–177	DRB1*0402	IFMGEI**EENSASNSL**VM	Dsg3 342–356	DRB1*0402	SVKL**SIAVKNKAE**FH
Dsg3 189–205	DRB1*0402	H**LNSKIAFKI**VSQEPAG	Dsg3 342–358	DRB1*0402	SVK**LSIAVKNKA**EFHQS
	DQB1*0503	H**LNSKIAFKI**VSQEPAG		DQB1*0503	SVKLS**IAVKNKAEF**HQS
Dsg3 190–204	DRB1*0402	LNS**KIAFKIVSQ**EPA	Dsg3 376–392	DQB1*0503	NVR**EGIAFRPAS**KTFTV
	DQB1*0503	LNS**KIAFKIVSQ**EPA	Dsg3 380–396	DRB1*0402	GIA**FRPASKTFT**VQKGI
Dsg3 191–205	DRB1*0402	NSKIA**FKIVSQEPA**G	Dsg3 483–499	DRB1*0402	VRVPD**FNDNCPTAV**LEK
Dsg3 205–221	DRB1*0402	GTPMFL**LSRNTGEVR**TL	Dsg3 762–776	DRB1*0402	QSGTM**RTRHSTGGT**N
	DQB1*0503	GTPMFLL**SRNTGEVRT**L	Dsg3 786–800	DRB1*0402	MNFLDS**YFSQKAFAC**
Dsg3 206–220	DRB1*0402	TPM**FLLSRNTGE**VRT	Dsg3 810–824	DRB1*0402	ND**CLLIYDNEG**ADAT
	DQB1*0503	TPMFL**LSRNTGEVR**T	Dsg3 963–977	DRB1*0402	ERVICP**ISSVPGNLA**
Dsg3 210–226	DRB1*0402	LLS**RNTGEVRTL**TNSL			

Preferred binding registers of experimentally determined Dsg3 peptides for PV implicated alleles. Best fitting registers with lowest predicted binding energies are in bold, underlined text.
