# Supplementary material for: Prediction of desmoglein-3 peptides reveals multiple shared T-cell epitopes in HLA DR4- and DR6- associated Pemphigus vulgaris
Source: BMC Bioinformatics. 2006 Dec 18;7(Suppl 5):S7. doi: 10.1186/1471-2105-7-S5-S7 (PMC1764484; doi:10.1186/1471-2105-7-S5-S7)
Supplement: Additional file 1 — Table-S1 [file 1471-2105-7-S5-S7-S1.pdf]

## SUPPLEMENTARY TABLES

**Table S1.** DRB1*0402-specific peptides with experimental $IC_{50}$ values used in this study.

| No. | Allele | Category | Description | Peptide | $IC_{50}$ (nM) | Reference |
|---|---|---|---|---|---|---|
| 1 | DRB1*0402 | Training Set | Dsg3 342-356 | SVKLSIAVKNKAEFH | 2600 | Sinha *et al.* (2006) |
| 2 | DRB1*0402 | Training Set | Dsg3 786-800 | MNFLDSYFSQKAFAC | 4700 | Sinha *et al.* (2006) |
| 3 | DRB1*0402 | Training Set | Dsg3 810-824 | NDCLLIYDNEGADAT | >40000 | Sinha *et al.* (2006) |
| 4 | DRB1*0402 | Training Set | Dsg3 67-81 | RNPIAKITSDYQATQ | >40000 | Sinha *et al.* (2006) |
| 5 | DRB1*0402 | Training Set | Dsg3 846-860 | LDSLGPKFKKLAEIS | 8900 | Sinha *et al.* (2006) |
| 6 | DRB1*0402 | Training Set | Dsg3 963-977 | ERVICPISSVPGNLA | >40000 | Sinha *et al.* (2006) |
| 7 | DRB1*0402 | Training Set | Dsg3 96-110 | PFGIFVVDKNTGDIN | >40000 | Sinha *et al.* (2006) |
| 8 | DRB1*0402 | Training Set | Dsg3 191-205 | NSKIAFKIVSQEPAG | 2700 | Sinha *et al.* (2006) |
| 9 | DRB1*0402 | Test Set | HADP analogue | AAVAAAKAAAAAA | 17 | Marshall *et al*. (1994) |
| 10 | DRB1*0402 | Test Set | HADP analogue | AAWAAAKAAAAAA | 2200 | Marshall *et al*. (1994) |
| 11 | DRB1*0402 | Test Set | HADP 7.18 | AAYAAAKAAALAA | 7000 | Marshall *et al*. (1994) |
| 12 | DRB1*0402 | Test Set | Myoglobin 110-122 | AIIHVLHSRHPGD | 1.9 | Marshall *et al*. (1994) |
| 13 | DRB1*0402 | Test Set | Myoglobin 67-79 | TVLTALGAILKKK | 640 | Marshall *et al*. (1994) |
| 14 | DRB1*0402 | Test Set | Myelin BP 90-102 | HFFKNIVTPRTPA | 61 | Marshall *et al*. (1994) |
| 15 | DRB1*0402 | Test Set | Tetanus toxoid 828-840 | MQYIKANSKFIGI | 900 | Marshall *et al*. (1994) |
| 16 | DRB1*0402 | Test Set | Pertussis Toxin 31-43 | NVLDHLTGRSSQV | 340 | Marshall *et al*. (1994) |
| 17 | DRB1*0402 | Test Set | Hemagglutinin 103-115 | PDYASLRSLVASS | 18 | Marshall *et al*. (1994) |
| 18 | DRB1*0402 | Test Set | Hemagglutinin 307-319 | PKYVKQNTLKLAT | 110 | Marshall *et al*. (1994) |
| 19 | DRB1*0402 | Test Set | *M. leprae* 65 kDa 416-428 | TLLQAAPALDKLK | 870 | Marshall *et al*. (1994) |
| 20 | DRB1*0402 | Test Set | HADP analogue | AAFAAAKAAAAAA | 47 | Marshall *et al*. (1994) |
| 21 | DRB1*0402 | Test Set | HADP analogue | AALAAAKAAAAAA | 2 | Marshall *et al*. (1994) |
| 22 | DRB1*0402 | Test Set | HADP analogue | AASAASKAAAAAA | 60000 | Marshall *et al*. (1994) |
| 23 | DRB1*0402 | Test Set | HADP 7.20 | AAYAAAKAAAVAA | 13000 | Marshall *et al*. (1994) |
| 24 | DRB1*0402 | Test Set | HADP 7.21 | AAYAAAKAAASAA | 4500 | Marshall *et al*. (1994) |
| 25 | DRB1*0402 | Test Set | HADP 7.44 | AAYAAAKAEAAAA | 3400 | Marshall *et al*. (1994) |
| 26 | DRB1*0402 | Test Set | HADP 18.7 | AAYAAAKAAAAAA | 1600 | Marshall *et al*. (1994) |
| 27 | DRB1*0402 | Test Set | HADP 7.25 | AAYAAAKAAAGAA | 5700 | Marshall *et al*. (1994) |
| 28 | DRB1*0402 | Test Set | HADP 7.45 | AAYAAAKALAAAA | 60 | Marshall *et al*. (1994) |
| 29 | DRB1*0402 | Test Set | HADP 7.50 | AAYAAFKAAAAAA | 460 | Marshall *et al*. (1994) |
| 30 | DRB1*0402 | Test Set | HADP 7.27 | AAYAAKKAAAAAA | 850 | Marshall *et al*. (1994) |
| 31 | DRB1*0402 | Test Set | HADP 7.30 | AAYAALKAAAAAA | 1900 | Marshall *et al*. (1994) |
| 32 | DRB1*0402 | Test Set | HADP 7.39 | AAYAAQKAAAAAA | 2700 | Marshall *et al*. (1994) |
| 33 | DRB1*0402 | Test Set | HADP 7.29 | AAYAASKAAAAAA | 5200 | Marshall *et al*. (1994) |
| 34 | DRB1*0402 | Test Set | Flu NP 383-395 | SRYWAIRTRSGGI | 13 | Marshall *et al*. (1994) |
| 35 | DRB1*0402 | Test Set | Matrix 18-30 | GPLKAEIAQRLED | 25000 | Marshall *et al*. (1994) |
| 36 | DRB1*0402 | Test Set | Tetanus toxoid 591-603 | KIYSYFPSVISKV | 9.2 | Marshall *et al*. (1994) |
| 37 | DRB1*0402 | Test Set | Haemagglutinin 23-35 | GTLVKTITDDQIE | 1200 | Marshall *et al*. (1994) |

**Table S1 (continued).** DRB1*0402-specific peptides with experimental $IC_{50}$ values used in this study.

| No. | Allele | Category | Description | Peptide | $IC_{50}$ (nM) | Reference |
|---|---|---|---|---|---|---|
| 38 | DRB1*0402 | Test Set | HADP 7.23 | AAYAAAKAAARAA | 4200 | Marshall *et al*. (1994) |
| 39 | DRB1*0402 | Test Set | HADP 7.46 | AAYAAAKAFAAAA | 200 | Marshall *et al*. (1994) |
| 40 | DRB1*0402 | Test Set | HADP 7.43 | AAYAAAKAKAAAA | 830 | Marshall *et al*. (1994) |
| 41 | DRB1*0402 | Test Set | HA Y307-319 | YPKFVKQNTLKAA | 2200 | Harfouch-Hammoud *et al*. (1999) |
| 42 | DRB1*0402 | Test Set | Hsp65 189-201 analogue | EGMRFAKGYISGY | 1000 | Hammer *et al*. (1995) |
| 43 | DRB1*0402 | Test Set | Designer peptide | GFKYAAAAAA | 6000 | Hammer *et al*. (1995) |
| 44 | DRB1*0402 | Test Set | Designer peptide | GFKAAARAAA | 9509 | Hammer *et al*. (1995) |
| 45 | DRB1*0402 | Test Set | Designer peptide | GFKAAAHAAA | 60000 | Hammer *et al*. (1995) |
| 46 | DRB1*0402 | Test Set | HLA-B | GRLLRGHNQFAYDGK | 5 | Kirschmann *et al*. (1995) |
| 47 | DRB1*0402 | Test Set | Synthetic peptide | DTQFVRFDSDAASQR | 600 | Kirschmann *et al*. (1995) |
| 48 | DRB1*0402 | Test Set | Apoliopoprotein | TPDFIVPLTDLRIPS | 70 | Kirschmann *et al*. (1995) |
| 49 | DRB1*0402 | Test Set | Actin peptide | YPIEHGIVTNWDDM | 4000 | Kirschmann *et al*. (1995) |
| 50 | DRB1*0402 | Test Set | Synthetic peptide | EEFVVEFDLPGIK | 100 | Kirschmann *et al*. (1995) |
| 51 | DRB1*0402 | Test Set | Synthetic peptide | AEFVVEFDLPGIK | 1000 | Kirschmann *et al*. (1995) |
| 52 | DRB1*0402 | Test Set | Synthetic peptide | EAFVVEFDLPGIK | 1000 | Kirschmann *et al*. (1995) |
| 53 | DRB1*0402 | Test Set | Synthetic peptide | EEFAVEFDLPGIK | 250 | Kirschmann *et al*. (1995) |
| 54 | DRB1*0402 | Test Set | Synthetic peptide | EEFVAEFDLPGIK | 250 | Kirschmann *et al*. (1995) |
| 55 | DRB1*0402 | Test Set | Synthetic peptide | EEFVVAFDLPGIK | 100 | Kirschmann *et al*. (1995) |
| 56 | DRB1*0402 | Test Set | Synthetic peptide | EEFVVEADLPGIK | 1000 | Kirschmann *et al*. (1995) |
| 57 | DRB1*0402 | Test Set | Synthetic peptide | EEFVVEFALPGIK | 400 | Kirschmann *et al*. (1995) |
| 58 | DRB1*0402 | Test Set | Synthetic peptide | EEFVVEFDAPGIK | 5500 | Kirschmann *et al*. (1995) |
| 59 | DRB1*0402 | Test Set | Synthetic peptide | EEFVVEFDLAGIK | 7000 | Kirschmann *et al*. (1995) |
