# Supplementary material for: Prediction of desmoglein-3 peptides reveals multiple shared T-cell epitopes in HLA DR4- and DR6- associated Pemphigus vulgaris
Source: BMC Bioinformatics. 2006 Dec 18;7(Suppl 5):S7. doi: 10.1186/1471-2105-7-S5-S7 (PMC1764484; doi:10.1186/1471-2105-7-S5-S7)
Supplement: Additional file 2 — Table-S2 [file 1471-2105-7-S5-S7-S2.pdf]

SUPPLEMENTARY TABLES

Table S2. Dsg3 peptides with experimental T-cell proliferation values/responses used in this study.

No.	Allele	Category	Description	Peptide	Reference
1	DRB1*0402	Test Set	Dsg3 342-358	SVKLSIAVKNKAEFHQS	Veldman *et al*. (2004)
2	DRB1*0402	Test Set	Dsg3 205-221	GTPMFLLSRNTGEVRTL	Veldman *et al*. (2004)
3	DRB1*0402	Test Set	Dsg3 380-396	GIAFRPASKTFTVQKGI	Riechers *et al*. (1999)
4	DRB1*0402	Test Set	Dsg3 190-204	LNSKIAFKIVSQEPA	Wucherpfennig *et al*. (1995)
5	DRB1*0402	Test Set	Dsg3 189-205	HLNSKIAFKIVSQEPAG	Veldman *et al*. (2004)
6	DRB1*0402	Test Set	Dsg3 512-526	SARTLNNRYTGPYTF	Wucherpfennig *et al*. (1995)
7	DRB1*0402	Test Set	Dsg3 78-94	QATQKITYRISGVGIDQ	Veldman *et al*. (2004)
8	DRB1*0402	Test Set	Dsg3 78-93	QATQKITYRISGVGID	Wucherpfennig *et al*. (1995)
9	DRB1*0402	Test Set	Dsg3 206-220	TPMFLLSRNTGEVRT	Wucherpfennig *et al*. (1995)
10	DRB1*0402	Test Set	Dsg3 210-226	LLSRNTGEVRTLTNSL	Veldman *et al*. (2004)
11	DRB1*0402	Test Set	Dsg3 251-265	CECNIKVKDVNDNFP	Wucherpfennig *et al*. (1995)
12	DRB1*0402	Test Set	Dsg3 250-266	QCECNIKVKDVNDNFPM	Veldman *et al*. (2004)
13	DRB1*0402	Test Set	Dsg3 483-499	VRVPDFNDNCPTAVLEK	Veldman *et al*. (2004)
14	DRB1*0402	Test Set	Dsg3 762-776	QSGTMRTRHSTGGTN	Wucherpfennig *et al*. (1995)
15	DRB1*0402	Test Set	Dsg3 161-177	IFMGEIEENSASNSLVM	Hertl *et al*. (1998)
16	DRB1*0402	Test Set	Dsg3 96-112	PFGIFVVDKNTGDINIT	Veldman *et al*. (2004)
17	DRB1*0402	Test Set	Dsg3 97-111	FGIFVVDKNTGDINI	Wucherpfennig *et al*. (1995)
18	DRB1*0402	Test Set	Dsg3 342-356	SVKLSIAVKNKAEFH	Moesta *et al*. (2002)
19	DRB1*0402	Test Set	Dsg3 846-860	LDSLGPKFKKLAEIS	Moesta *et al*. (2002)
20	DRB1*0402	Test Set	Dsg3 67-81	RNPIAKITSDYQATQ	Moesta *et al*. (2002)
21	DRB1*0402	Test Set	Dsg3 786-800	MNFLDSYFSQKAFAC	Moesta *et al*. (2002)
22	DRB1*0402	Test Set	Dsg3 191-205	NSKIAFKIVSQEPAG	Moesta *et al*. (2002)
23	DRB1*0402	Test Set	Dsg3 96-110	PFGIFVVDKNTGDIN	Moesta *et al*. (2002)
24	DRB1*0402	Test Set	Dsg3 963-977	ERVICPISSVPGNLA	Moesta *et al*. (2002)
25	DRB1*0402	Test Set	Dsg3 810-824	NDCLLIYDNEGADAT	Moesta *et al*. (2002)
26	DQB1*0503	Training Set	Dsg3 342-358	SVKLSIAVKNKAEFHQS	Veldman *et al*. (2004)
27	DQB1*0503	Training Set	Dsg3 376-392	NVREGIAFRPASKTFTV	Veldman *et al*. (2004)
28	DQB1*0503	Training Set	Dsg3 205-221	GTPMFLLSRNTGEVRTL	Veldman *et al*. (2004)
29	DQB1*0503	Training Set	Dsg3 250-266	QCECNIKVKDVNDNFPM	Veldman *et al*. (2004)
30	DQB1*0503	Training Set	Dsg3 96-112	PFGIFVVDKNTGDINIT	Veldman *et al*. (2004)
31	DQB1*0503	Training Set	Dsg3 512-526	SARTLNNRYTGPYTF	Wucherpfennig *et al*. (1995)
32	DQB1*0503	Training Set	Dsg3 97-111	FGIFVVDKNTGDINI	Wucherpfennig *et al*. (1995)
33	DQB1*0503	Training Set	Dsg3 78-93	QATQKITYRISGVGID	Wucherpfennig *et al*. (1995)
34	DQB1*0503	Test Set	Dsg3 78-94	QATQKITYRISGVGIDQ	Veldman *et al*. (2004)
35	DQB1*0503	Test Set	Dsg3 189-205	HLNSKIAFKIVSQEPAG	Veldman *et al*. (2004)
36	DQB1*0503	Test Set	Dsg3 762-786	QSGTMRTRHSTGGTN	Wucherpfennig *et al*. (1995)
37	DQB1*0503	Test Set	Dsg3 190-204	LNSKIAFKIVSQEPA	Wucherpfennig *et al*. (1995)
38	DQB1*0503	Test Set	Dsg3 206-220	TPMFLLSRNTGEVRT	Wucherpfennig *et al*. (1995)
39	DQB1*0503	Test Set	Dsg3 251-265	CECNIKVKDVNDNFP	Wucherpfennig *et al*. (1995)
